# Suboptimal quality of female oncofertility care is associated with lowered quality of life

**DOI:** 10.1007/s11136-026-04213-z

**Published:** 2026-04-01

**Authors:** M. van den Berg, F. Lusing, C. C. M. Beerendonk, S. E. J. Kaal, C. M. P. W. Mandigers, T. N. Schuurman, J. Tol, J. M. Tromp, M. J. D. L. van der Vorst, D. D. M. Braat, R. P. M. G. Hermens

**Affiliations:** 1 Department of Obstetrics and Gynecology, Radboud university medical center, Nijmegen, The Netherlands; 2 Department of Medical Oncology, Radboud University Medical Center, Nijmegen, The Netherlands; 3 Dutch AYA ‘Young and Cancer’ Care Network, IKNL, Utrecht, The Netherlands; 4 Department of Medical Oncology, Canisius-Wilhelmina Hospital, Nijmegen, The Netherlands; 5 Center for Gynecologic Oncology Amsterdam, Cancer Institute-Antoni van Leeuwenhoek Hospital, Amsterdam, The Netherlands; 6 Department of Medical Oncology, Jeroen Bosch Hospital, Den Bosch, The Netherlands; 7 Department of Medical Oncology, Amsterdam University Medical Center, Amsterdam, The Netherlands; 8 Department of Medical Oncology, Rijnstate Hospital, Arnhem, The Netherlands; 9 Department of IQ Health, Radboud University Medical Center, P.O. Box 9101, Nijmegen, 6500 HB the Netherlands

**Keywords:** Oncofertility, Female cancer survivors, Quality of life, Decisional conflict, Decision regret, Quality of care

## Abstract

**Purpose:**

Female adolescent and young adult cancer patients should be informed about infertility risks due to cancer treatment. However, quality of female oncofertility care is far from optimal. It is not known whether this suboptimal quality of care is associated with patient-reported outcome measures (PROMs), particularly quality of life, decisional conflict, regret, and reproductive concerns.

**Methods:**

A multicenter cross-sectional survey study was conducted to measure the association between quality of oncofertility care and PROMs in female cancer survivors from six hospitals across the Netherlands. Quality indicators were used to assess quality of care, and validated scales to assess PROMs. Quality indicator and PROM scores were calculated, and possible associated determinants were analyzed by *T*-tests and multilevel multivariate analyses.

**Results:**

Female cancer survivors (*N* = 121) received suboptimal quality of care with 8 out of 11 quality indicators scoring < 90% adherence. When survivors were informed about infertility risks, were offered fertility preservation counseling, and received digital/written information, their physical quality of life was highest (51.05 vs. 44.05), and levels of decisional conflict (24.55 vs. 48.22) and regret (12.50 vs. 28.67) were lowest, compared to non-adherence. Quality of life and decision regret were significantly influenced by relationship status, strength of wish to conceive, and type of cancer.

**Conclusion:**

Receiving high-quality integrated female oncofertility care is associated with an improved quality of life, and with less decisional conflict and regret in female cancer survivors. As quality of oncofertility care is suboptimal, tailored strategies should be developed to guideline-specific barriers, to improve quality of care and, most importantly, quality of life in female cancer survivors.

**Supplementary Information:**

The online version contains supplementary material available at 10.1007/s11136-026-04213-z.

## Introduction

 The importance of addressing the late side effects of cancer treatments and long-term quality of life issues has increased since cancer survival rates in female adolescent and young adult (AYA) cancer patients have improved [1–4] and cancer incidence has risen globally [5]. Female AYA cancer patients prefer a ‘normal’ life after surviving cancer, in which fertility and the possibility to have children are key components [6, 7]. Therefore, clinical guidelines recommend oncological healthcare professionals to provide information about infertility risks and fertility preservation (FP) options, and to offer a referral to and counseling by a reproductive gynecologist [8–11]. Unfortunately, studies have shown that adherence to these guidelines is suboptimal, with not all patients being informed about infertility risks or offered FP counseling [12–17].

Previous studies have shown that female cancer survivors’ quality of life is lower, and decision regret is higher when they did not receive FP counseling [18, 19]. Furthermore, decisional conflict increases when survivors have unmet information needs or negative experiences with FP counseling, and decision regret is higher when they did not have FP treatment [20–23]. Besides receiving FP counseling and having FP treatment, more key elements are important in delivering high-quality integrated female oncofertility care. In our previous study we selected key recommendations from international guidelines and transcribed them into 11 quality indicators (QIs) [24, 25]. QIs were distributed over four domains, particularly, risk communication by the oncological healthcare provider, referral to a gynecologist for FP counseling, FP counseling, and shared decision-making on FP^24^. It has been demonstrated that quality of integrated female oncofertility care measured with these QIs is far from optimal [13].

As mentioned, previous studies reported the impact of FP counseling and additional information on Patient-Reported Outcome Measures (PROMs), such as quality of life and decision regret. However, it is not yet known whether adherence to oncofertility guidelines measured with QIs of all four domains is associated with a better quality of life and FP knowledge, less decisional conflict, regret, and reproductive concerns in female AYA cancer survivors. In a few studies in cancer care, but not yet in oncofertility care, it has been proven that adhering to guidelines was associated with better health-related quality of life among cancer survivors [26–28]. Furthermore, insight into determinants which influence patient-reported outcomes as quality of life should be taken into account when developing tailored improvement strategies.

Within this study, our first aim was therefore to measure the association between quality of integrated female oncofertility care measured with QIs and quality of life, decisional conflict, decision regret, reproductive concerns, and FP knowledge in female AYA cancer survivors. We hypothesize that more adherence to QIs will increase quality of life and FP knowledge and decrease decisional conflict, decision regret and reproductive concerns. Our second aim was to measure which determinants were associated with these PROMs to be able to develop tailored improvement strategies.

## Methods

### Design and setting

This multicenter cross-sectional study was conducted by means of a survey to female AYA cancer survivors in six Dutch hospitals. In the Netherlands, patients receive multidisciplinary oncological care and can be referred for FP counseling by any healthcare provider involved. Patients have no financial reasons to refrain from FP counseling or FP treatment, because it is covered by basic health insurance, or by the hospital cryobanks itself. This study was approved by the Medical Ethics Committee of Arnhem-Nijmegen (NL61570.091.17).

### Study population

Female AYA cancer survivors (18–40 years) who were diagnosed in 2016 or 2017 and received gonadotoxic treatment were eligible to participate. The Netherlands Cancer Registry was used by the Netherlands Comprehensive Cancer Organization to identify potentially eligible survivors. The patients’ primary oncological healthcare provider was asked to assess the following exclusion criteria: patient was deceased, severely diseased, had severe psychological problems, had undergone a hysterectomy and/or oophorectomy before the start of cancer treatment, or did not receive follow-up care in the participating hospital. The oncological healthcare provider assessed patient’s eligibility for study participation, determining whether they could be invited based on the presence of psychological conditions, such as depressive or anxiety disorders.

### Survey development

The survey consisted of baseline and clinical characteristics, questions that represented the QIs (answered with yes/no), and survey questions that represented PROMs. We sought to minimize potential bias by incorporating validated survey. No changes were made to items from these validated surveys. Crohnbach’s alfa reliability tests were performed for survey scales, α > 0.80 indicated internal consistency. For FP knowledge no reliability was analysed.

### Survey outcome measures

#### Quality indicators

A total of 40 questions was developed to measure the 11 QIs in female oncofertility care in our previous studies [13, 24]. Supplementary Table 1 shows all QIs.

#### Quality of life

Quality of life was measured using the validated 12-item Short Form Health Survey (SF-12) [29]. The summary physical and mental SF-12 scores were based on the scoring algorithms manual with higher scores representing a better quality of life and scores below 50 a lowered quality of life [30]. A thermometer in which survivors had to score their current health status on a scale of 0-100 was added by our research team. The thermometer captures an overall subjective evaluation of current health status and corresponds to the EuroQol 5 Dimensions (Eq. 5D) visual analogue scale. It complements the SF-12 by offering a singe, intuitive rating of perceived health, while adding minimal respondent burden.

#### Decisional conflict

Female cancer survivor’s decisional conflict was measured using a validated Dutch translation of the Decisional Conflict Scale [31]. This scale includes 16 items, evaluating how certain survivors felt about their decision on FP treatment on a five-point Likert scale, ranging from 0(totally disagree) to 4(totally agree) [32]. Item 15 (‘I expect to stick with my decision’) was removed due to the retrospective design of our study. Items were converted to a score of 0–100 with higher scores representing higher levels of conflict. Scores below 25 represent absence of decisional conflict, whereas scores exceeding 37.5 are associated with decision delay and feeling unsure [32].

#### Decision regret

Female cancer survivor’s current regret regarding their past decision on FP was measured using the validated Decision Regret Scale on a five-point Likert scale, ranging from 1(totally disagree) to 5(totally agree) [33]. Items were converted to a score of 0 (absent regret) – 100 (extreme regret) and interpreted as follows: 0, no regret; 1–25, mild regret; and > 25, moderate to severe regret [33].

#### Reproductive concerns

Reproductive concerns were measured using a validated Dutch translation of the Reproductive Concerns Scale consisting of 11 items scored on a five-point Likert scale ranging from 0(not at all) to 4(very much) [34]. The items include thoughts and feelings related to fertility, reproduction or having children. Total score ranged from 0 to 44, with higher scores representing more reproductive concerns. For healthy controls the mean score is 6.4^34^.

#### Fertility preservation knowledge

FP knowledge was measured using 12 statements which were retrieved from two questionnaires from previous studies [35, 36]. Answering categories were ‘true’, ‘false’ or ‘do not know’. A total FP knowledge score was calculated (range 0–12), with a higher score representing more knowledge.

### Data collection

Eligible female cancer survivors received an invitation letter from their primary oncological healthcare provider to participate in 2020/2021. After obtaining written informed consent, a survey was sent by e-mail. If no informed consent or completed survey was received within three weeks, one reminder was sent. To reduce selection bias, all eligible participants were invited to participate using identical recruitment procedures.

### Data analysis

QI adherence scores were calculated in our previous study [13]. PROMs were analyzed descriptively according to the published survey manuals. If missing rates are more than 10%, imputations will be performed.

To evaluate which determinants were associated with PROMs, we first studied the univariate relation between PROMs (dependent variables) and determinants (independent variables) that could influence the PROM. Determinants were extracted from literature and included age, relationship status, parity, strength of wish to conceive, type of cancer, and treatment, time before start of cancer treatment, type of healthcare provider, and hospital [14, 15, 37]. We used a generalized linear mixed model which accounts for the nested structure of data, because individual patients (patient level = 1) are nested within hospitals (hospital level = 2). Multivariate linear regression analyses were performed for determinants with *p* < 0.20 in univariate analyses. A *p*-value threshold of < 0.20 in univariate analyses was used to identify candidate determinants for inclusion in the multivariate models, in order to avoid premature exclusion of potentially relevant variables. Determinants with *p* < 0.05 were considered significant.We tested for collinearity between determinants. If a correlation (> 0.6) was detected between determinants, the most relevant variable with respect to content was included in the multivariate analyses.

Independent samples T-tests were used to evaluate whether QI adherence scores were associated with the PROMs, only for those with a minimum of 10 responses. Thereafter, multivariate analyses were performed to adjust for the effect of the determinants that were associated with the PROMs. Furthermore, QIs were combined for adherence to evaluate if (non)-adherence to a combination of QIs was associated with PROMs. Again, a minimum of 10 responses was needed to develop a reliable multilevel model.

## Results

### Study population

In total, 566 survivors were identified of whom 344 met inclusion criteria. Main reasons for exclusion by their primary health care provider were: survivor did not receive follow-up care in participating hospital(*N* = 77), had severe psychological problems(*N* = 35), or was severely diseased(*N* = 30). A total of 150 out of 344 survivors provided informed consent of whom 121(35%) returned their survey. Mean female cancer survivor’s age at diagnosis was 34 years, and 37.2% had FP treatment (Table 1).

**Table 1:** Baseline characteristics

Characteristic	Number (%)
Response rate	121/344 (35)
Mean age at diagnosis	34.0 years (range 18–41, SD 5.1)
Type of cancer	
Bladder cancer	1 (0.8)
Brain tumor	2 (1.7)
Breast cancer	73 (60.3)
Cervical cancer	14 (11.6)
Colorectal cancer	5 (4.1)
Head and neck cancer	1 (0.8)
Leukemia	3 (2.5)
Lung cancer	1 (0.8)
Lymphoma	15 (12.4)
Osteosarcoma	2 (1.7)
Ovarian cancer	2 (1.7)
Soft tissue sarcoma	2 (1.7)
Cancer treatment	
Breast surgery	72 (59.5)
Chemotherapy	112 (92.6)
Hormone therapy	45 (37.2)
Immunotherapy	20 (16.5)
Irradiation therapy	80 (66.1)
Breast	− 56 (70)
Skull/craniospinal	− 8 (10)
Pelvic	− 10 (12.5)
Total body	− 6 (7.5)
Uterine or ovarian surgery	11 (9.1)
Relationship status at diagnosis	
In a relationship	110 (90.9)
Single	11 (9.1)
Parity	
Nulliparous	50 (41.3)
Parous	71 (58.7)
Strength of wish to conceive at diagnosis (mean, scale 1–10)	5.8 (range 1–10, SD 3.5)
Number of survivors who received fertility preservation counseling	62/121 (51.2)
Number of survivors who had fertility preservation treatment (3 survivors had 2 treatments)	45/121 (37.2)
Oocyte cryopreservation	24 (53.3)
Embryo cryopreservation	11 (24.4)
Ovarian tissue cryopreservation	3 (6.7)
Trachelectomy or unilateral ovariectomy	9 (20)
GnRH agonists	1 (2.2)

### Quality indicator scores

Guideline adherence, measured with the 11 QIs, is presented in Table 2. Patients received suboptimal quality of care with 8 out of 11 QIs scoring < 90% adherence. Off all survivors, 72.7% was informed about their infertility risks, 51.2% was offered FP counseling, with 18.8% all aspects were discussed in FP counseling, and 35.5% received written/digital information. Two patients did not respond to the question whether SDM has been made (QI10). No other missing data on QIs was reported.

**Table 2:** Quality indicator scores

Quality indicator	Total score Nominator/denominator	%
QI1—Risk of infertility discussed within 2 weeks after diagnosis		
Yes	88/121	72.7
No	33/121	
QI2—Risk of infertility discussed when pelvic, skull, craniospinal or total body irradiation takes place		
Yes	23/24	95.8
No	1/24	
QI3—Oncological healthcare provider consulted expert colleague when he/she had insufficient knowledge about fertility preservation		
Yes	7/16	43.8
No	9/16	
QI4—Opportunity of counseling with a gynecologist is offered		
Yes	62/121	51.2
No	59/121	
QI5—Individualized selection and risk analysis has been executed within an expert multidisciplinary team prior to fertility preservation treatment		
Yes	19/21	90.5
No	2/21	
QI6—Oocyte and embryo cryopreservation has been discussed with eligible patients		
Yes	22/22	100
No	0/22	
QI7—Embryo cryopreservation is offered as an effective and safe method, when time and circumstances allow for it		
Yes	34/39	87.2
No	5/39	
QI8—All aspects (a–h) have been discussed in fertility preservation counseling		
Yes	6/32	18.8
No	26/32	
QI9—Well-informed about all aspects of the treatment prior to performing emergency IVF		
Yes	27/33	81.8
No	6/33	
QI10—Shared decision has been made concerning protecting future fertility		
Yes	44/60	73.3
No	16/60	
QI11—Decision was supported with written and/or digital information		
Yes	43/121	35.5
No	78/121	

^a^Two patients did not respond to the question whether shared decision has been made

### Determinants associated with patient-reported outcome measures

Multivariate analyses showed that four determinants at patient level (age, relationship status, strength of wish to conceive, and type of cancer) influenced the scores of physical quality of life, decision regret, reproductive concerns, and FP knowledge significantly (Table 3). A higher wish to conceive was associated with a higher physical quality of life. Survivors who were single at diagnosis or who had another type of cancer than breast cancer had higher levels of decision regret. Furthermore, higher levels of reproductive concerns were associated with survivors who were younger, were single and had a higher wish to conceive.

**Table 3:** Mean scores of patient-reported outcome measures and their associated determinants in multilevel multivariate analyses

Patient-reported outcome measure	Mean score (range) (SD)	Determinants	*N*	Multilevel multivariate analysis Coefficient (CI), *p*-value
Physical quality of life (scale 0-100)	48.47	Strength of wish to conceive		
	(28.13–62.48)	1–5	61	3.33 (0.20–6.47) p0.038Ref
	(8.84)	6–10	60	
Mental quality of life (scale 0-100)	47.95	None significant		
	(21.08–60.94)			
	(9.80)			
Current health status (scale 0-100)	74.98	None significant		
	(20–100)			
	(12.81)			
Decisional conflict (scale 0-100)	31.22	None significant		
	(0–98.33)			
	(19.73)			
Decision regret (scale 0-100)	19.10	Relationship status		
	(0–90)	Single	10	2.81 (0.15–5.48) p0.039Ref
	(21.63)	Partner	111	
		Type of cancer		
		Breast cancer	73	1.66 (− 0.001 to 3.32) p0.05Ref
		Other than breast cancer	48	
Reproductive concerns (scale 0–44)	11.21	Female cancer survivor’s age		
	(0–44)	18–29	24	5.88 (0.99–10.78) p0.019Ref
	(11.27)	30–41	97	
		Relationship status		
		Single	10	8.06 (1.36–14.76) p0.019Ref
		Partner	111	
		Strength of wish to conceive		
		1–5	61	9.49 (5.77–13.22) p0.000Ref
		6–10	60	
Fertility preservation knowledge (scale 0–12)	8.91	Female cancer survivor’s age		
	(1–12)	18–29	24	0.89 (0.03–1.76) p0.044Ref
	(1.90)	30–41	97	
		Strength of wish to conceive		
		1–5	61	0.89 (0.21–1.58) p0.011Ref
		6–10	60	

### Patient-reported outcome measures and their association with guideline adherence

Results of the mean PROM scores of all female cancer survivors are shown in Table 3. Complete case analysis was performed since missing data was less than 10%. All validated scale surveys were internally consistent (α > 0.80). Adherence to the QI and PROMs was calculated for four out of 11 QIs (QI1, 4, 10, 11) and are shown in Table 4. Three QIs could be combined for adherence (QI1, 4 and 11).

**Table 4:** Patient-reported outcome measures and their association with fertility preservation counseling, treatment, and quality indicator adherence

Patient-reported outcome measure	Physical quality of life^4^ (scale 0-100) Mean score *p*-value	Mental quality of life (scale 0-100) Mean score *p*-value	Current health status (scale 0-100) Mean score *p*-value	Decisional conflict (scale 0-100) Mean score *p*-value	Decision regret^2^ (scale 0-100) Mean score *p*-value	Reproductive concerns^3^ (scale 0–44) Mean score *p*-value	FP knowledge^1^ (scale 0–12) Mean score *p*-value
Received FP counseling (N = 62)	51.06	49.33	79.06	26.47	15.57	14.67	9.69
Did not receive FP counseling (N = 59)	45.76	46.61	70.68	36.60	23.40	7.76	8.16
*T*-test (*p*-value)	**0**.**001**	0.114	**0**.**000**	**0**.**006**	0.058	**0**.**001**	**0**.**000**
Multivariate analyses (*p*-value)	**0**.**007**				**0**.**011**	0.524	**0**.**002**
Had FP treatment (N = 45)	50.36	50.05	77.58	22.54	12.44	13.29	9.92
Did not have FP treatment (N = 76)	47.36	47.36	73.43	36.92	23.64	10.09	8.39
*T*-test (*p*-value)	0.071	0.070	0.085	**0**.**000**	**0**.**007**	0.159	**0**.**000**
Multivariate analyses (*p*-value)	0.283				**0**.**002**	0.343	**0**.**002**
Association with adherence to quality indicator							
QI1—Infertility risks discussed							
Yes (N = 88)	48.82	48.20	75.76	27.94	17.38	11.11	9.01
No (N = 33)	47.52	47.29	72.87	40.06	23.97	11.48	8.62
*T*-test (*p*-value)	0.471	0.652	0.272	**0**.**004**	0.160	0.881	0.341
Multivariate analyses (*p*-value)	0.531				0.143	0.590	0.513
QI4—Opportunity of counseling with gynecologist is offered							
Yes (N = 62)	51.18	49.98	78.92	26.83	18.07	12.81	9.36
No (N = 59)	45.62	45.82	70.83	36.37	20.41	9.54	8.47
*T*-test (*p*-value)	**0**.**000**	**0**.**019**	**0**.**000**	**0**.**011**	0.573	0.132	**0**.**012**
Multivariate analyses (*p*-value)	**0**.**002**				0.431	0.656	0.195
QI10—Shared decision concerning FP has been made							
Yes (N = 44)	51.06	50.35	78.38	23.37	14.31	15.54	9.76
No (N = 16)	50.29	46.45	80.19	34.79	20.00	13.38	9.73
*T*-test (*p*-value)	0.736	0.169	0.553	**0**.**022**	0.310	0.550	0.949
Multivariate analyses (*p*-value)	0.451				0.358	0.606	0.823

Patient-reported outcome measure	Physical quality of life^1^ (scale 0-100) Mean score *p*-value	Mental quality of life (scale 0-100) Mean score *p*-value	Current health status (scale 0-100) Mean score *p*-value	Decisional conflict (scale 0-100) Mean score *p*-value	Decision regret^2^ (scale 0-100) Mean score *p*-value	Reproductive concerns^**3**^ (scale 0–44) Mean score *p*-value	FP knowledge^4^ (scale 0–12) Mean score *p*-value
QI11—Decision was supported with written and/or digital information							
Yes (N = 43)	50.23	49.64	78.23	24.22	11.34	10.94	9.70
No (N = 78)	47.50	47.02	73.18	35.63	23.64	11.35	8.47
*T*-test (*p*-value)	0.104	0.161	**0**.**037**	**0**.**003**	**0**.**003**	0.860	**0**.**001**
Multivariate analyses (*p*-value)	0.208				**0**.**005**	0.357	**0**.**004**
Association with adherence to quality indicators combined							
QI1 + QI4							
Adherence to both (N = 49)	50.80	50.23	78.84	26.45	18.47	12.41	9.46
Non-adherence to both (N = 20)	44.20	46.16	68.75	49.12	27.31	9.35	8.50
*T*-test (*p*-value)	**0**.**002**	0.111	**0**.**003**	**0**.**000**	0.073	0.334	**0**.**036**
Multivariate analyses (*p*-value)	**0**.**006**				0.144	0.732	0.164
QI1 + QI11							
Adherence to both (N = 35)	50.15	48.72	78.69	23.67	11.32	11.24	9.79
Non-adherence to both (N = 25)	46.53	45.25	71.80	44.92	27.96	12.10	8.41
*T*-test (*p*-value)	0.104	0.206	0.062	**0**.**000**	**0**.**003**	0.800	**0**.**002**
Multivariate analyses (*p*-value)	0.128				**0**.**002**	0.269	**0**.**008**
QI4 + QI11							
Adherence to both (N = 32)	51.28	49.96	80.28	23.13	13.37	11.81	9.86
Non-adherence to both (N = 48)	45.26	45.16	70.50	38.83	23.33	9.77	8.28
*T*-test (*p*-value)	**0**.**004**	**0**.**039**	**0**.**002**	**0**.**001**	**0**.**023**	0.484	**0**.**001**
Multivariate analyses (*p*-value)	**0**.**011**				0.052	0.600	**0**.**016**
QI1 + QI4+QI11							
Adherence to all three (N = 26)	51.05	48.98	80.92	24.55	12.50	12.62	9.96
Non-adherence to all three (N = 18)	44.05	45.49	68.33	48.22	28.67	8.93	8.44
*T*-test (*p*-value)	**0**.**006**	0.287	**0**.**005**	**0**.**000**	**0**.**005**	0.356	**0**.**004**
Multivariate analyses (*p*-value)	**0**.**014**				**0**.**016**	0.173	**0**.**012**

FP: fertility preservation, QI: quality indicator. ^1^: multivariate analyses to adjust effect for strength of wish to conceive, ^2^: multivariate analyses to adjust effect for relationship status and type of cancer, ^3^: multivariate analyses to adjust effect for female cancer survivor’s age, relationship status, and strength of wish to conceive, ^4^: multivariate analyses to adjust effect for female cancer survivor’s age and strength of wish to conceive

Exploratory note on scales: DRS scores > 25 represents moderate to severe regret. DCS scores < 25 represent absence of decisional conflict, scores > 37.5 are associated with decision delay and feeling unsure. SF-12 scores < 50 represent a lowered quality of life. The mean score for healthy controls of the RCS is 6.4

#### Quality of life

Physical and mental quality of life was slightly lower than in the average healthy population (48.47 and 47.95 vs. 50). Female cancer survivors who received FP counseling had significant higher levels of physical quality of life. Even when only the opportunity of counseling was offered, higher levels of physical and mental quality of life were reported. Physical quality of life was lowest when survivors were not informed about infertility risks, were not offered counseling, and did not receive written/digital information (44.05 vs. 51.05 for adherence). Regarding mental quality of life, greatest significant difference was seen between adherence and non-adherence to QI4 (FP counseling offered) and QI11 (received written/digital information) (49.96 vs. 45.16).

Female cancer survivors scored their current health status 74.98 on a scale of 0–100. This score significantly increased when they were offered FP counseling or received written/digital information. Again, score was highest when there was adherence to all three QIs (80.92 vs. 68.33 for non-adherence).

#### Decisional conflict

Decisional conflict was perceived by female cancer survivors, with a mean score of 31.22. Levels of decisional conflict significantly increased when there was no adherence to all QIs separately, as well as for all combinations of QIs. When female cancer survivors were not informed about infertility risks, were not offered counseling, and received no written/digital information, decisional conflict levels exceeded far above 37.5 (score of 48.22) which is associated with decision delay and feeling unsure.

#### Decision regret

Decision regret levels significantly decreased when female cancer survivors received FP counseling (15.57 vs. 23.40), had FP treatment (12.44 vs. 23.64), or received written/digital information (13.37 vs. 23.33). The highest level of decision regret was found when there was no adherence to all three QIs (28.67 vs. 12.50 for adherence).

#### Reproductive concerns

Mean score of reproductive concerns was 11.21 on a scale of 0–44. For all QIs, levels of reproductive concerns did not differ significantly.

#### Fertility preservation knowledge

Overall, female cancer survivors had a high FP knowledge score. Survivors who received FP counseling, had FP treatment, or received written/digital information reported significantly more FP knowledge. Again, FP knowledge was highest when there was adherence to all three QIs.

## Discussion

Receiving high-quality integrated female oncofertility care measured with QIs is associated with improved quality of life and FP knowledge, and with less decisional conflict and regret in female AYA cancer survivors. When there was adherence to three QIs (i.e. survivors were informed about infertility risks, were offered FP counseling, and received digital/written information), meaning receiving higher quality of oncofertility care, female cancer survivors’ quality of life and FP knowledge was highest, and levels of decisional conflict and regret were lowest. In addition, four determinants distracted from literature (female cancer survivors’ age, relationship status, strength of wish to conceive, and type of cancer) were found to significantly influence physical quality of life, decision regret, reproductive concerns, and FP knowledge.

To the best of our knowledge, this is one of the first studies which demonstrated that adhering to Qis, and therefore adhering to international guidelines, in integrated female oncofertility care is associated with an improved quality of life in female AYA cancer survivors. In line with our study, an improved quality of life was seen in female cancer survivors who received FP counseling or FP treatment in previous studies [18, 19]. Not only FP treatment, but FP counseling itself could increase quality of life, since FP counseling also supports patients with the emotional distress associated with potential infertility at the time of cancer diagnosis, and infertility in survivorship [38]. In addition, we demonstrated that offering FP counseling and adherence to more QIs was associated with an improved physical quality of life. Some studies have shown that decisional conflict increases when female cancer survivors have unmet information needs [22, 23]. We were able to confirm these results. A way to meet information needs and to decrease decisional conflict is the provision of a FP decision aid [39]. Our research team developed a tailored FP decision aid, however its effect on decisional conflict is still to be evaluated [40].

In contrast to a previous study, we did not find an association between receiving FP counseling and higher levels of reproductive concerns after adjusting for the associated determinants [41]. Difference in outcome might be explained by the higher number of survivors who received counseling (141 vs. 62 in our study), and because the reproductive concerns after cancer (RCAC) scale was used (vs. RCS in our study). The RCS focuses primarily on generalized feelings about fertility loss, and lacks the multidimensional structure that captures the nuanced concerns documented among AYA cancer survivors which are mentioned in the RCAC scale [42] (e.g., worries about passing on a genetic predisposition to cancer, child health, personal health and recurrence of cancer). Therefore, the use of the unidimensional RCS may underrepresent the diversity of concerns that are affected by cancer and loss of fertility.

All above mentioned studies focused on one or two elements in female oncofertility care. To the contrary, we focused on all domains in integrated female oncofertility care, particularly on information provision, offering referral for FP counseling, FP counseling by a gynecologist, and FP decision-making. All domains have shown to be important in delivering high-quality care as these were selected as key elements by a multidisciplinary expert panel [24]. Within our previous study we showed that quality of oncofertility care is far from optimal on all these domains and not only on receiving information or FP counseling [13].

It is often assumed that suboptimal guideline adherence, hence a suboptimal quality of care, is associated with a decreased quality of life. However, only a few previous studies, all not in female oncofertility care, were able to prove this assumption. An American study demonstrated that quality of elderly female cancer survivors’ life improved when adherence to the guideline cancer prevention recommendations was higher [28]. Also, a Dutch study showed that adherence to these recommendations improved quality of life among colorectal cancer survivors, and a Chinese study showed this association among breast cancer patients [26, 27]. With our study, we added evidence to the assumption that a suboptimal quality of integrated oncofertility care is associated with a lowered quality of life.

A strength of our study is that it is the first study that showed an association between quality of integrated female oncofertility care and quality of life, decisional conflict, and decision regret in cancer survivors. In contrast to other studies, we analyzed more PROMs, and analyzed their association with adherence to all domains that have proven to be important in delivering high-quality integrated female oncofertility care.

There are also a few limitations to our study. First, selection bias could have occurred as we have no insight into reasons for non-responding and we have a low response rate (35%), although this rate is in line with previous studies among AYA cancer survivors [43]. Perceived oncofertility care and PROM results might be different for non-responders than for responders, which might have biased our results. Second, because of the low response rate, we could not analyze the association with PROMs for all QIs. Furthermore, we could not perform multivariate analyses for the association between QIs and decisional conflict as there were no significant influencing determinants. However, associations were highly significant between QI scores and decisional conflict in *T*-tests. Selection bias could have underestimated PROMs. Survey responders might differ from non-responders in distress levels, engagement, or fertility concerns, potentially underestimating decisional conflict and regret and overestimating quality of life. Last, recall bias could have occurred in outcome measures as patients were asked to fill in questions three to four years after their diagnosis, which may have led survivors with high regret or decisional conflict to underreport care received, and survivors with better quality of life to overreport care.

Despite these limitations, the findings of our study are likely to be generalizable to AYA cancer survivors in similar healthcare settings, as our sample reflects a range of diagnoses and treatment contexts. Nevertheless, caution is warranted when extrapolating these results to populations with different demographic or healthcare characteristics. Notably, in countries with more limited access to or coverage of FP services, patients may experience higher decisional conflict, greater regret, increased reproductive concerns, and potentially lower quality of life, as opportunities for timely counseling and treatment are constrained.

Several conclusions and implications for clinical practice can be drawn from our study. As we have shown that suboptimal quality of integrated female oncofertility care is associated with a lower quality of life in female cancer survivors, improvement strategies tailored to the current gaps in oncofertility care and to guideline-specific barriers should be developed and thereafter implemented [44]. Examples of improvement strategies include standardized written/digital information, such as a decision aid, structured referral pathways, education of professionals and a role for patient fertility navigators and specialized oncology nurses [45]. At this moment, some improvement strategies have already been implemented. Described effects are positive, with more documentations on infertility risks, more access to FP counseling, and more satisfaction with received information [46–49]. However, a systematic evaluation of all aspects of integrated quality of female oncofertility care and PROMs is lacking. In future studies, our QI set could be used internationally to evaluate whether newly developed tailored strategies have a positive effect on quality of integrated female oncofertility care, and, most importantly, on quality of life in female cancer survivors.

## Supplementary Information

Below is the link to the electronic supplementary material.

Supplementary Material 1

## Data Availability

Study data are available upon request.

## References

[1] De Angelis, R., Demuru, E., Baili, P., et al. (2024). Complete cancer prevalence in Europe in 2020 by disease duration and country (EUROCARE-6): A population-based study. *The Lancet Oncology*, *25*(3), 293–307. 10.1016/S1470-2045(23)00646-0 38307102 10.1016/S1470-2045(23)00646-0

[2] Aben, K. K., van Gaal, C., van Gils, N. A., van der Graaf, W. T., & Zielhuis, G. A. (2012). Cancer in adolescents and young adults (15–29 years): a population-based study in the Netherlands 1989–2009. *Acta Oncologica*, *51*(7), 922–933. 10.3109/0284186x.2012.705891 22934554 10.3109/0284186X.2012.705891

[3] Robison, L. L., & Hudson, M. M. (2014). Survivors of childhood and adolescent cancer: Life-long risks and responsibilities. *Nature Reviews Cancer*, *14*(1), 61–70. 10.1038/nrc3634 24304873 10.1038/nrc3634 PMC6425479

[4] Miller, K. D., Fidler-Benaoudia, M., Keegan, T. H., Hipp, H. S., Jemal, A., & Siegel, R. L. (2020). Cancer statistics for adolescents and young adults, 2020. *C Ca: A Cancer Journal For Clinicians*, *70*(6), 443–459. 10.3322/caac.21637 10.3322/caac.21637 32940362

[5] Bray, F., Ferlay, J., Soerjomataram, I., Siegel, R. L., Torre, L. A., & Jemal, A. (2018). Global cancer statistics 2018: GLOBOCAN estimates of incidence and mortality worldwide for 36 cancers in 185 countries. *C Ca: A Cancer Journal For Clinicians*, *68*(6), 394–424. 10.3322/caac.21492 10.3322/caac.21492 30207593

[6] Armuand, G. M., Wettergren, L., Rodriguez-Wallberg, K. A., & Lampic, C. (2015). Women more vulnerable than men when facing risk for treatment-induced infertility: a qualitative study of young adults newly diagnosed with cancer. *Acta Oncologica*, *54*(2), 243–252. 10.3109/0284186x.2014.948573 25140859 10.3109/0284186X.2014.948573

[7] Logan, S., Perz, J., Ussher, J. M., Peate, M., & Anazodo, A. (2018). Systematic review of fertility-related psychological distress in cancer patients: Informing on an improved model of care. *Psychooncology*. 10.1002/pon.4927 30460732 10.1002/pon.4927

[8] Lambertini, M., Peccatori, F. A., Demeestere, I., et al. (2020). Fertility preservation and post-treatment pregnancies in post-pubertal cancer patients: ESMO clinical practice guidelines (dagger). *Annals of Oncology*, *31*(12), 1664–1678. 10.1016/j.annonc.2020.09.006 32976936 10.1016/j.annonc.2020.09.006

[9] NVOG. (2016). Fertility Preservation for women with cancer. https://richtlijnendatabase.nl/richtlijn/fertiliteitsbehoud_bij_vrouwen_met_kanker/fertiliteitsbehoud_vrouwen_met_kanker_-_startpagina.html

[10] Anderson, R. A., Amant, F., Braat, D., et al. (2020). ESHRE guideline: Female fertility preservation. *Hum Reprod Open*, *2020*(4), hoaa052. 10.1093/hropen/hoaa052 33225079 10.1093/hropen/hoaa052 PMC7666361

[11] Oktay, K., Harvey, B. E., Partridge, A. H., et al. (2018). Fertility preservation in patients with cancer: ASCO clinical practice guideline update. *Journal of Clinical Oncology*, *36*(19), 1994–2001. 10.1200/jco.2018.78.1914 29620997 10.1200/JCO.2018.78.1914

[12] Anazodo, A., Laws, P., Logan, S., et al. (2019). How can we improve oncofertility care for patients? A systematic scoping review of current international practice and models of care. *Human Reproduction Update*, *25*(2), 159–179. 10.1093/humupd/dmy038 30462263 10.1093/humupd/dmy038 PMC6390168

[13] van den Berg, M., Kaal, S. E. J., Schuurman, T. N., et al. (2023). Quality of integrated female oncofertility care is suboptimal: A patient-reported measurement. *Cancer Medicine*, *12*(3), 2691–2701. 10.1002/cam4.5149 36031940 10.1002/cam4.5149 PMC9939180

[14] Bastings, L., Baysal, O., Beerendonk, C. C., Braat, D. D., & Nelen, W. L. (2014). Referral for fertility preservation counselling in female cancer patients. *Human Reproduction*, *29*(10), 2228–2237. 10.1093/humrep/deu186 25069500 10.1093/humrep/deu186

[15] Skaczkowski, G., White, V., Thompson, K., et al. (2018). Factors influencing the provision of fertility counseling and impact on quality of life in adolescents and young adults with cancer. *Journal Of Psychosocial Oncology*, *36*(4), 484–502. 10.1080/07347332.2018.1443986 29764330 10.1080/07347332.2018.1443986

[16] Salsman, J. M., Yanez, B., Smith, K. N., et al. (2016). Documentation of fertility preservation discussions for young adults with cancer: Examining compliance with treatment guidelines. *Journal of the National Comprehensive Cancer Network*, *14*(3), 301–309. 26957616 10.6004/jnccn.2016.0035 PMC5537734

[17] Pathak, S., Vadaparampil, S. T., Sutter, M. E., Rice, W. S., & McBride, C. M. (2023). Evaluating fertility preservation interventions for alignment with ASCO Guidelines for reproductive aged women undergoing cancer treatment: A systematic review. *Supportive Care In Cancer*, *31*(12), 689. 10.1007/s00520-023-08133-3 37950073 10.1007/s00520-023-08133-3 PMC10638151

[18] Benedict, C., Thom, B., Friedman, D. N., Pottenger, E., Raghunathan, N., & Kelvin, J. F. (2018). Fertility information needs and concerns post-treatment contribute to lowered quality of life among young adult female cancer survivors. *Supportive Care In Cancer*. 10.1007/s00520-017-4006-z 29387996 10.1007/s00520-017-4006-z PMC5984121

[19] Deshpande, N. A., Braun, I. M., & Meyer, F. L. (2015). Impact of fertility preservation counseling and treatment on psychological outcomes among women with cancer: A systematic review. *Cancer*. 10.1002/cncr.29637 26264701 10.1002/cncr.29637

[20] Benedict, C., Thom, B., & Kelvin, J. F. (2015). Young adult female cancer survivors’ decision regret about fertility preservation. *Journal of Adolescent and Young Adult Oncology**4*(4), 213–218. 10.1089/jayao.2015.0002 26697271 10.1089/jayao.2015.0002 PMC4684663

[21] Chan, J. L., Letourneau, J., Salem, W., et al. (2017). Regret around fertility choices is decreased with pre-treatment counseling in gynecologic cancer patients. *Journal of Cancer Survivorship*, *11*(1), 58–63. 10.1007/s11764-016-0563-2 27480882 10.1007/s11764-016-0563-2 PMC5269477

[22] Bastings, L., Baysal, Ö., Beerendonk, C. C., et al. (2014). Deciding about fertility preservation after specialist counselling. *Human Reproduction*, *29*(8), 1721–1729. 10.1093/humrep/deu136 24916435 10.1093/humrep/deu136

[23] Benedict, C., & Thom, B. (2016). Young adult female cancer survivors’ unmet information needs and reproductive concerns contribute to decisional conflict regarding posttreatment fertility preservation. *Cancer*, *122*(13), 2101–2109. 10.1002/cncr.29917 27213483 10.1002/cncr.29917 PMC4911318

[24] Baysal, Ö., Van den Berg, M., Beldman, F., et al. (2020). Key recommendations for high-quality female oncofertility care based on international clinical practice guidelines. *Reproductive Biomedicine Online*, *40*(3), 409–422. 10.1016/j.rbmo.2019.11.016 32057675 10.1016/j.rbmo.2019.11.016

[25] Baysal, O., Hamilton, J. A. M., Hamilton, C., Braat, D. D. M., Beerendonk, C. C. M., & Nelen, W. (2018). Clinical practice guidelines for fertility preservation in young women undergoing gonadotoxic treatment: An overview and critical appraisal of methodological quality and content. *Reproductive Biomedicine Online*, *37*(1), 60–70. 10.1016/j.rbmo.2018.03.022 29709394 10.1016/j.rbmo.2018.03.022

[26] van Veen, M. R., Mols, F., Bours, M. J. L., Weijenberg, M. P., Kampman, E., & Beijer, S. (2019). Adherence to the World Cancer Research Fund/American Institute for Cancer Research recommendations for cancer prevention is associated with better health-related quality of life among long-term colorectal cancer survivors: Results of the PROFILES registry. *Supportive Care In Cancer*, *27*(12), 4565–4574. 10.1007/s00520-019-04735-y 30927111 10.1007/s00520-019-04735-y PMC6825038

[27] Lei, Y. Y., Ho, S. C., Cheng, A., et al. (2018). Adherence to the World Cancer Research Fund/American Institute for Cancer Research guideline is associated with better health-related quality of life among Chinese patients with breast cancer. *Journal of the National Comprehensive Cancer Network*, *16*(3), 275–285. 10.6004/jnccn.2017.7202 29523666 10.6004/jnccn.2017.7202

[28] Inoue-Choi, M., Lazovich, D., Prizment, A. E., & Robien, K. (2013). Adherence to the World Cancer Research Fund/American Institute for Cancer Research recommendations for cancer prevention is associated with better health-related quality of life among elderly female cancer survivors. *Journal of Clinical Oncology*, *31*(14), 1758–1766. 10.1200/jco.2012.45.4462 23569318 10.1200/JCO.2012.45.4462 PMC3641697

[29] Gandek, B., Ware, J. E., Aaronson, N. K., et al. (1998). Cross-validation of item selection and scoring for the SF-12 Health Survey in nine countries: results from the IQOLA Project. International Quality of Life Assessment. *Journal of Clinical Epidemiology*, *51*(11), 1171–1178. 10.1016/s0895-4356(98)00109-7 9817135 10.1016/s0895-4356(98)00109-7

[30] Ware, J., John, E., Keller, S. D., & Kosinski, M. (1995). SF-12: How to score the SF-12 physical and mental health summary scales. *Health Institute New England Medical Center*.

[31] Koedoot, N., Molenaar, S., Oosterveld, P., et al. (2001). The decisional conflict scale: Further validation in two samples of Dutch oncology patients. *Patient Education And Counseling*, *45*(3), 187–193. 10.1016/s0738-3991(01)00120-3 11722854 10.1016/s0738-3991(01)00120-3

[32] O’Connor, A. M. (1995). Validation of a decisional conflict scale. *Medical Decision Making*, *15*(1), 25–30. 10.1177/0272989X9501500105 7898294 10.1177/0272989X9501500105

[33] Brehaut, J. C., O’Connor, A. M., Wood, T. J., et al. (2003). Validation of a decision regret scale. *Medical Decision Making*, *23*(4), 281–292. 10.1177/0272989x03256005 12926578 10.1177/0272989X03256005

[34] Garvelink, M. M., ter Kuile, M. M., Louwe, L. A., Hilders, C. G., & Stiggelbout, A. M. (2015). Validation of a Dutch version of the reproductive concerns scale (RCS) in three populations of women. *Health Care For Women International*, *36*(10), 1143–1159. 10.1080/07399332.2014.993036 25562531 10.1080/07399332.2014.993036

[35] Balthazar, U., Deal, A. M., Fritz, M. A., Kondapalli, L. A., Kim, J. Y., & Mersereau, J. E. (2012). The current fertility preservation consultation model: Are we adequately informing cancer patients of their options? *Human Reproduction*, *27*(8), 2413–2419. 10.1093/humrep/des188 22674206 10.1093/humrep/des188 PMC6457080

[36] Peate, M., Meiser, B., Friedlander, M., et al. (2011). It’s now or never: Fertility-related knowledge, decision-making preferences, and treatment intentions in young women with breast cancer—An Australian fertility decision aid collaborative group study. *Journal of Clinical Oncology*, *29*(13), 1670–1677. 10.1200/jco.2010.31.2462 21444865 10.1200/JCO.2010.31.2462

[37] Jones, G., Hughes, J., Mahmoodi, N., Smith, E., Skull, J., & Ledger, W. (2017). What factors hinder the decision-making process for women with cancer and contemplating fertility preservation treatment? *Human Reproduction Update*, *23*(4), 433–457. 10.1093/humupd/dmx009 28510760 10.1093/humupd/dmx009

[38] Logan, S., & Anazodo, A. (2019). The psychological importance of fertility preservation counseling and support for cancer patients. *Acta Obstetricia et Gynecologica Scandinavica*, *98*(5), 583–597. 10.1111/aogs.13562 30723914 10.1111/aogs.13562

[39] Wang, Y., Anazodo, A., & Logan, S. (2019). Systematic review of fertility preservation patient decision aids for cancer patients. *Psychooncology*, *28*(3), 459–467. 10.1002/pon.4961 30523651 10.1002/pon.4961

[40] van den Berg, M., van der Meij, E., Bos, A. M. E., et al. (2021). Development and testing of a tailored online fertility preservation decision aid for female cancer patients. *Cancer Medicine*, *10*(5), 1576–1588. 10.1002/cam4.3711 33580749 10.1002/cam4.3711 PMC7940215

[41] Young, K., Shliakhtsitsava, K., Natarajan, L., et al. (2019). Fertility counseling before cancer treatment and subsequent reproductive concerns among female adolescent and young adult cancer survivors. *Cancer*, *125*(6), 980–989. 10.1002/cncr.31862 30489638 10.1002/cncr.31862 PMC6402962

[42] Gorman, J. R., Pan-Weisz, T. M., Drizin, J. H., Su, H. I., & Malcarne, V. L. (2019). Revisiting the reproductive concerns after cancer (RCAC) scale. *Psychooncology*, *28*(7), 1544–1550. 10.1002/pon.5130 31128074 10.1002/pon.5130 PMC8428791

[43] Kaal, S. E. J., Husson, O., van Duivenboden, S., et al. (2017). Empowerment in adolescents and young adults with cancer: Relationship with health-related quality of life. *Cancer*, *123*(20), 4039–4047. 10.1002/cncr.30827 28696580 10.1002/cncr.30827 PMC5655905

[44] Grol, R. W. M., & Eccles, M. (2005). Improving patient care. The implementation of change in clinical practice.

[45] van den Berg, M., Baysal, O., Nelen, W., Braat, D. D. M., Beerendonk, C. C. M., & Hermens, R. (2019). Professionals’ barriers in female oncofertility care and strategies for improvement. *Human Reproduction*, *34*(6), 1074–1082. 10.1093/humrep/dez062 31111876 10.1093/humrep/dez062

[46] Lewin, J., Ma, J. M., Mitchell, L., et al. (2017). The positive effect of a dedicated adolescent and young adult fertility program on the rates of documentation of therapy-associated infertility risk and fertility preservation options. *Supportive Care In Cancer*. 10.1007/s00520-017-3597-8 28155019 10.1007/s00520-017-3597-8

[47] Vu, J. V., Llarena, N. C., Estevez, S. L., Moravek, M. B., & Jeruss, J. S. (2017). Oncofertility program implementation increases access to fertility preservation options and assisted reproductive procedures for breast cancer patients. *Journal of Surgical Oncology*, *115*(2), 116–121. 10.1002/jso.24418 27966219 10.1002/jso.24418 PMC5292230

[48] Bradford, N. K., Walker, R., Henney, R., Inglis, P., & Chan, R. J. (2017). Improvements in clinical practice for fertility preservation among young cancer patients: Results from bundled interventions. *Journal of Adolescent and Young Adult Oncology*. 10.1089/jayao.2017.0042 28934554 10.1089/jayao.2017.0042

[49] Kelvin, J. F., Thom, B., Benedict, C., et al. (2016). Cancer and fertility program improves patient satisfaction with information received. *Journal of Clinical Oncology*, *34*(15), 1780–1786. 10.1200/jco.2015.64.5168 27044937 10.1200/JCO.2015.64.5168 PMC4966338

