# Supplementary material for: Suboptimal quality of female oncofertility care is associated with lowered quality of life
Source: Qual Life Res. 2026 Apr 1;35(5):120. doi: 10.1007/s11136-026-04213-z (PMC13043604; doi:10.1007/s11136-026-04213-z)
Supplement: Supplementary file 1 — Supplementary Material 1 [file 11136_2026_4213_MOESM1_ESM.docx]

**Supplementary Table 1. Quality indicators distributed over the domains in female oncofertility care**

| **Domain** | **Quality indicators** |
| --- | --- |
| **Risk**  **Communication**  **QI1**  **QI2**  **QI3** | Percentage of patients in an oncological process who receive a gonadotoxic treatment... |
|  | with whom at least the risk of infertility is discussed by their oncological healthcare provider early in the oncological process (i.e. within 2 weeks). |
|  | with whom the consequences (of the treatment) for their fertility are discussed when they have a wish to conceive, and when their ovaries, uterus, hypothalamus, pituitary gland (or all) are in the irradiation area (pelvic, skull, craniospinal irradiation or total body irradiation). |
|  | of whom the oncological healthcare provider consulted an expert colleague when he/she had insufficient knowledge about fertility preservation. |
| **Referral QI4** | Percentage of patients in an oncological process who receive a gonadotoxic treatment to whom the opportunity of counseling with a gynecologist with expertise in fertility preservation is offered. |
| **Counseling**  **QI5**  **QI6**  **QI7**  **QI8**  **QI9** | Percentage of patients in an oncological process who receive a gonadotoxic treatment... |
|  | for whom an individualized selection and risk analysis has been executed within an expert multidisciplinary team (i.e. primary oncological healthcare provider and treating gynecologist). |
|  | (who are estimated as medically fit for the procedure, are expected to be able to tolerate the treatment regimen, have sufficient time before the commencement of their cancer treatment and, are informed of the potential risks of hormonal treatment including the risks of cancer progression), with whom oocyte and embryo cryopreservation has been discussed during fertility preservation counseling. |
|  | to whom embryo cryopreservation is offered as an effective and safe method, when time and circumstances allow for it. |
|  | who have had fertility preservation counseling with a gynecologist in which all aspects (a – h) have been discussed.  a. The chance to preserve ovarian, uterine function, or both, and the chance of spontaneous pregnancy after cancer treatment.  b. The chance to preserve ovarian, uterine function, or both, and the chance of pregnancy when using different fertility preservation methods, and expectations for the future.  c. The risks of fertility preservation procedures: delay of cancer treatment, surgery (laparoscopy, laparotomy), risk of reintroducing the tumor (metastases) after autotransplantation of cryopreserved ovarian tissue, premature menopause after cancer treatment and unilateral and partial oophorectomy.  d. The conditions to undergo fertility treatment after cancer treatment, (number of years a patient should be relapse-free after curation, posthumous reproduction, etc.). The contracts should also be discussed.  e. Alternatives, such as oocyte donation, gestational surrogacy or adoption.  f. Necessary tests before a fertility preservation treatment, such as standard screening for viral pathogens and sexually transmitted diseases.  g. Hormonal screening through blood testing.  h. Possibilities to treat endocrine consequences owing to the loss of ovarian function. |
|  | who have been well-informed about all aspects of the treatment prior to performing emergency IVF. |
| **Decision-making**  **QI10**  **QI11** | Percentage of patients in an oncological process who receive a gonadotoxic treatment... |
|  | with whom a shared decision has been made concerning protecting future fertility (together with oncological healthcare provider and gynecologist) |
|  | who have had fertility preservation counseling which was supported with written, digital information, or both. |
